# Clinical and diagnostic characteristics of non-alcoholic fatty liver disease among Egyptian children and adolescents with type1 diabetes

**DOI:** 10.1186/s13098-023-01029-6

**Published:** 2023-03-21

**Authors:** Hanaa Reyad Abdallah, Eman Refaat Youness, Manar Maher Bedeir, Marwa W. Abouelnaga, Wafaa M. Ezzat, Yasser Elhosary, Hazem Mohamed El-Hariri, Mona Abd Elmotaleb A. Hussein, Heba R. Ahmed, Rasha Eladawy

**Affiliations:** 1 Biological Anthropology Department, Medical Research and Clinical Studies Institute, National Research Centre, Cairo, Egypt; 2 Medical Biochemistry Department, Medical Research and Clinical Studies Institute, National Research Centre, Cairo, Egypt; 3 Child Health Department, Medical Research and Clinical Studies Institute, National Research Centre, Cairo, Egypt; 4 Internal Medicine Department, Medical Research and Clinical Studies Institute, National Research Centre, Cairo, Egypt; 5 Community Medicine Department, Medical Research and Clinical Studies Institute, National Research Centre, Cairo, Egypt; 6 National Institute of Diabetes and Endocrinology, Cairo, Egypt; 7 Faculty of Medicine, Ain Shams University, Cairo, Egypt

**Keywords:** Type1 diabetes, Hepatopathy, Non-alcoholic fatty liver disease (NAFLD), Anthropometric measures, Acoustic radiation force impulse elastography (ARFI)

## Abstract

**Background:**

Type 1 diabetes mellitus (T1DM) patients are at an increased risk for non-alcoholic fatty liver disease (NAFLD). This study aimed to evaluate the clinical criteria associated with the diagnosis of Non-Alcoholic Fatty Liver Disease (NAFLD) among T1DM Egyptian children and adolescents.

**Methods:**

74 T1DM patients aged 8–18 year were enrolled in this cross sectional study. Assessments of Clinical status, anthropometric measures, lipid profile, glycated haemoglobin (HbA1c) and liver enzymes were done. Abdominal Ultrasound evaluation of hepatic steatosis was done. Accordingly, patients were divided into two groups (NAFLD and normal liver group) and compared together. Assessment of liver fibrosis using acoustic radiation force impulse elastography (ARFI) was done. Statistical analysis included; independent t-test, Chi square and Fisher’s Exact, Pearson and Spearman tests and Logistic regression models for factors associated with fatty liver were used when appropriate.

**Results:**

In this study; 74 patients were enrolled; 37 males (50%) and 37 females with mean age 14.3 ± 3.0 year. The mean insulin dose was 1.1 ± 0.4 U/kg and mean disease duration was 6.3 ± 3.0 year. NAFLD was detected in 46 cases while 28 cases had normal liver as diagnosed by abdominal ultrasound. Cases with NAFLD had statistically significant higher BMI-Z scores, waist/hip, waist/height and sum of skin fold thicknesses compared to those with normal liver (P < 0.05). The mean value of HbA1c % was significantly higher in NAFLD group (P = 0.003). Total cholesterol, triglycerides and LDL serum levels were significantly elevated (p < 0.05), while the HDL level was significantly lower in NAFLD cases (p = 0.001). Although, serum levels of liver enzymes; ALT and AST were significantly higher among cases with NAFLD than in normal liver group (p < 0.05), their means were within normal. Using the ARFI elastography; NAFLD cases exhibited significant fibrosis (F2, 3 and 4). BMI, patient age and female gender were among risk factors for NAFLD.

**Conclusions:**

NAFLD represents a serious consequence in type 1 diabetic children and adolescents that deserves attention especially with poor glycemic control. NAFLD has the potential to evolve to fibrosis. This study demonstrated a very high prevalence of NAFLD in T1D children and adolescents using US which was (62.2%) with the percent of liver fibrosis among the NAFLD cases (F2-F4) using ARFI elastography was 26%. BMI, age of patients and female gender were detected as risk factors for NAFLD.

## Background

Diabetes mellitus type 1 (T1DM), is an autoimmune disease represents a serious, long-term condition with a major impact on the lives. The International Diabetes Federation (IDF) has reported more than 1 million children and adolescents suffer type 1 diabetes [1].

Non-alcoholic fatty liver disease (NAFLD) is the most common chronic liver disease among pediatric population and adults. Chronic liver diseases comprise a varied spectrum of diseases starting from simple steatosis or NAFLD, then non-alcoholic steatohepatitis (NASH), to cirrhosis. NAFLD should be diagnosed only when other causes of hepatic affection are absent [2, 3]. Throughout the last years, NAFLD has represented an apparently medical and financial burden as a result of increase in obesity and diabetes mellitus prevalence [4]. Furthermore, increased mortality caused by liver diseases was attributed to increase in NAFLD cases. [5]. Putting into consideration, that type 1 diabetes is a lifelong disease with extended duration, the range of NAFLD and its long standing consequences are clinically related to type 1 diabetic cases [6]. Inadequate excretion of triglycerides from the liver by VLD lipoproteins or hyperglycemia stimulates hepatic lipogenesis leading to accumulation of fats in the liver in type 1 diabetes mellitus (T1DM) [7]. Liver biopsy is the ‘‘gold standard’’ for the diagnosis of NAFLD. Nevertheless, it exposes patient to hazards of complications as it is an invasive technique [8]. The European guidelines for managing Non-Alcoholic Fatty Liver endorsed the usage of U/S as the first-line imaging in patients with possible Non-Alcoholic Fatty Liver and Non-Alcoholic Steato-Hepatitis [9]. In adults, its sensitivity is 90% and specificity is 95% for moderate to severe steatosis identification, however its sensitivity is decreased if the amount of liver fat is decreased than 33% [10]. In children, hepatic ultrasound can detect fat with 70–85% sensitivity and 50–60% specificity [11].

Histological characteristics have shown significantly more severe steatosis in pediatric NAFLD in comparison to adults [12]. When the disease progresses to severe fibrotic phase it is called pediatric non-alcoholic steatohepatitis (NASH) [13]. Accordingly, the necessity for precise diagnosis and staging of this disease is of great significance in people with high risk like pediatric diabetic population using non-invasive methods [14].

Pediatric patients with laboratory measures within normal levels, as well those with normal hepatic U/S or mild steatosis, could actually have had a considerable stage of hepatic fibrosis. Acoustic Radiation Force Impulse Imaging (ARFI) is a promising U/S -based technique for assessing hepatic fibrosis and stiffness with diagnostic accuracy comparable to that of Transient elastography (TE) and could be utilized as a non-invasive method to diagnose pediatric NAFLD particularly in subjects where biopsy is not a preferred technique [15, 16]. So far, there is a paucity in paediatric studies examining the association between liver disease and type 1 DM in children and adolescents.

## Methods

### Aim of the study

In this study, we aim to investigate the clinical and diagnostic characteristics distinguishing NAFLD associated with type1 diabetes in a cohort of type 1 diabetic Egyptian children and adolescents; using laboratory examination, ultrasound and liver stiffness measurement; acoustic radiation force imaging [ARFI].

### Study design and setting of the study

We conducted a cross sectional observational study enrolled 74 patients with type 1 diabetes, aged 8–18 years, and duration of T1DM > 2 years who were treated and followed in the National Institute of Diabetes and Endocrine Diseases and recruited to the outpatient clinic in the Medical Research Centre of Excellence in National Research Centre during the period from 2019 to 2021. Cases were further subdivided into two groups: patients with NAFLD and patients without NAFLD.

### Participants

Patients’ inclusion criteria included; (8–18) year, confirmed diagnosis of T1DM I according to International Society for Paediatric and Adolescent Diabetes (ISPAD) guidelines [17] and duration of T1DM > than 2 years.

Exclusion criteria included; patients who had a known other system affection like central nervous system, respiratory, renal, cardiovascular and congenital diseases or other endocrine disease like thyroid disorders, Mauriac syndrome, history of chronic liver diseases including history suggestive of viral hepatitis A, B, or C infection, genetic disorders, Wilson’s disease, hemochromatosis, and autoimmune hepatitis, and history of use of drugs causing liver function abnormality (hepatotoxic drugs such as tamoxifen, amiodarone, valproate and methotrexate or prednisone).

### Assessment of participants’ characteristics

All the study cases were subjected to the following; thorough history taking laying stress on; age of onset of diabetes, diabetes duration, diabetes complications, any long term medications other than insulin, diabetic ketoacidosis comas, insulin regimen treatment including; the type and dose of insulin in units (morning and night dose) then the mean of insulin units was taken, glycemic control as indicated by both HbA1C in the last 12 months, frequency of hypoglycemia in the last month (number of times the patient had blood glucose levels ≤ 70 mg/dL, associated diseases, and hospitalization due to any cause in the last year. In addition to gastrointestinal or hepatic symptoms like abdominal pain, nausea, or vomiting, jaundice, pruritus, visceral pain and abdominal distention. Family history of type 1 or 2 Diabetes, obesity and hypertension was also taken.

Complete clinical examination was performed stressing on; signs of hepatic affection including; jaundice, spider naevi, tender liver, hepatomegaly, splenomegaly, and ascites.

Blood pressure was measured according to American Heart Association guidelines; during the patients’ visits to the outpatient clinic, with the use of mercury sphygmomanometer; three consecutive blood pressures were measured for all patients with at least 5 min intervals, in a seated position and through a standard method using an appropriate cuff and sphygmomanometer. Blood pressure (BP) measurements were compared to age-specific percentiles for BP.

Anthropometric assessment was done as follows; Height and weight were measured. The body mass index (BMI) was calculated as weight (in kilograms) divided by height (in square meters). The standard deviation scores (SDS) of BMI were calculated using the WHO ANTHRO Plus softwares [18]. The waist circumference and hip circumference were measured. Waist/Hip ratio and Waist/Height ratio were calculated. The skinfold thicknesses were measured to the nearest 1.0 mm using Holtain skin fold caliber. They included; triceps, biceps, subscapular, suprailiac and abdominal skin folds. Each measurement was taken as the mean of three consecutive measurements, using standardized equipment and following the recommendations of International Biological programs [19].

### Laboratory and biochemical investigations

HbA1c was assessed. In addition, the mean of three readings of glycosylated hemoglobin HbA1c measurements during the last year for each patient was calculated to be representative of long-term metabolic control and patient was considered with poor glycemic control if > 10% regardless of age.

Fasting blood glucose was assessed using enzymatic colorimetric methods using a Hitachi auto analyzer 704 (Roche Diagnostics. Switzerland) [20].

### The diagnosis of NAFLD in our study was based on using these routine noninvasive evaluation including

1. *Biochemical parameters which included* complete lipid profile (serum total cholesterol, triglycerides, HDL, LDL) and liver enzymes; aspartate aminotransferase [AST], alanine aminotransferase [ALT] were carried out using automated clinical chemistry analyzer. HBVs Ag and HCV Ab were done using the PRECHECK Kits (USA). Serum Anti smooth muscle antibodies ASMA, Anti-nuclear antibody ANA and anti-liver and kidney microsomal antibodies LKM were measured using ELISA.
2. *Abdominal ultrasonography* which is the most commonly used imaging modality because it is relatively inexpensive, widely available to detect fatty liver. A routine liver ultrasound was performed by experienced radiologist. Examinations were performed according to a standardized protocol. US evaluation of hepatic steatosis typically consisted of a qualitative visual assessment of hepatic echogenicity, measurements of the difference between the liver and kidneys in echo amplitude, evaluation of echo penetration into the deep portion of the liver, and determination of the clarity of blood vessel structures in the liver. All US was performed by one of the two radiologists involved in the study who were blinded to the blood test-results and clinical history of patients.

### Measurement of liver fibrosis

*Acoustic Radiation Force Impulse elastography (ARFI) was done as follows*

Acoustic radiation force impulse elastography (ARFI) was performed for all subjects with a Siemens Acuson S3000 Virtual Touch ultrasound system (Siemens AG, Erlangen, Germany) with a 6CI transducer. The principle underlying ARFI elastography is that sharing of the examined tissue induces a strain in the tissues. An acoustic “push” pulse is automatically produced by the ultrasound probe and directed to the side of a region of interest (ROI), which is where the speed of the shear wave is measured. The acoustic “push” pulse generates shear waves that propagate into the tissue, perpendicular to the “push” axis. Detection waves are also generated by the transducer to measure the propagation speed of these shear waves, which increases with fibrosis severity [21]. For each patient, 10 valid ARFI measurements were performed under fasting conditions, with the patient in supine position with the right arm in maximum abduction, by the intercostal approach in the right liver lobe, 1–2 cm under the liver capsule. Minimal scanning pressure was applied, and the patient was asked to stop normal breathing for a moment to minimize breathing motion. The mean of 8–10 valid measurements was calculated and considered indicative of the severity of fibrosis.

### Statistical analysis

The collected data were coded, tabulated, and statistically analyzed using IBM SPSS statistics (Statistical Package for Social Sciences) software version 22.0, IBM Corp., Chicago, USA, 2013. Quantitative normally distributed data were described as mean ± SD (standard deviation) after testing for normality using Shapiro–Wilk test, then compared using independent t-test for normally distributed. While Pearson test was used for correlations of normally distributed data and Spearman correlation for ordinal data. Qualitative data were described as number and percentage and compared using Chi square test and Fisher’s Exact test for variables with small expected numbers. Logistic regression models were done for factors associated with fatty liver.

## Results

A total of 74 participants with type 1 diabetes mellitus were enrolled in this study where 37 (50%) were males and 37 were females (50%). The mean age of the studied cases was 14.3 ± 3.0 (8–18) year. The mean age of onset of diabetes was 8.0 ± 3.2 while the mean insulin dose/day was 1.1 ± 0.4 U/kg/day, and the mean disease duration was 6.3 ± 3.0 years. 94.6% of our cases had history of previous DKA attacks, 64.9% had symptoms of diabetic complications and 68.9% had symptoms of liver affection. Demographic data, clinical characteristics of the total studied cases and comparison according to U/S diagnosis of NAFLD are shown in (Tables 1, 2)

**Table 1:** Demographic characteristics among the studied cases and comparison according to the presence of NAFLD

Variables		All cases (N = 74)	NAFLD		P-value
			Present (N = 46)	Absent (N = 28)	
Age (years), mean ± SD		14.3 ± 3.0	15.4 ± 2.3	12.4 ± 3.1	^ < 0.001*
Gender (n, %)	Male	37 (50.0%)	17 (37.0%)	20 (71.4%)	#0.004*
	Female	37 (50.0%)	29 (63.0%)	8 (28.6%)	
Family history	Type 2 DM	47 (63.5%)	31 (67.4%)	16 (57.1%)	#0.374
	Obesity	33 (44.6%)	23 (50.0%)	10 (35.7%)	#0.231
	Liver disease	17 (23.0%)	13 (28.3%)	4 (14.3%)	#0.166
	Hypertension	30 (40.5%)	19 (41.3%)	11 (39.3%)	#0.864
	Consanguinity	13 (17.6%)	7 (15.2%)	6 (21.4%)	§0.539

*BMI* body mass index

^Independent t-test

^#^Chi square test

^§^Fishers exact test

^*^Significant (p < 0.050)

**Table 2:** DM characteristics among the studied cases and comparison according to the presence of NAFLD

Variables		All cases (N = 74)	NAFLD		P-value
			Present (N = 46)	Absent (N = 28)	
Age of onset (years), mean ± SD		8.0 ± 3.2	8.7 ± 3.0	6.8 ± 3.2	^0.015*
Duration (years), mean ± SD		6.3 ± 3.0	6.7 ± 3.2	5.6 ± 2.4	^0.110
Insulin dose (unit/kg/day) Mean ± SD		1.1 ± 0.4	1.1 ± 0.4	1.1 ± 0.3	^0.520
History of DKA (n, %)		70 (94.6%)	45 (97.8%)	25 (89.3%)	§0.149
DKA frequency (attack/duration), mean ± SD		0.7 ± 0.6	0.7 ± 0.5	0.6 ± 0.6	^0.458
Complications, (n, %)	Total cases with complications	48 (64.9%)	35 (76.1%)	13 (46.4%)	#0.010*
	Lipodystrophy	25 (33.8%)	20 (43.5%)	5 (17.9%)	#0.024*
	Joint affection	25 (33.8%)	19 (41.3%)	6 (21.4%)	#0.080
	Neuropathy	22 (29.7%)	18 (39.1%)	4 (14.3%)	#0.023*
	Nephropathy	7 (9.5%)	6 (13.0%)	1 (3.6%)	§0.242
Symptoms of liver affection, (n, %)	Total cases with symptoms	51 (68.9%)	37 (80.4%)	14 (50.0%)	#0.006*
	Abdominal pain	45 (60.8%)	33 (71.7%)	12 (42.9%)	#0.014*
	Nausea	30 (40.5%)	24 (52.2%)	6 (21.4%)	#0.009*
	Vomiting	24 (32.4%)	19 (41.3%)	5 (17.9%)	#0.037*
	Pruritis	10 (13.5%)	7 (15.2%)	3 (10.7%)	§0.733
	Jaundice	0 (0.0%)	0 (0.0%)	0 (0.0%)	Not applicable

^Independent t-test

^#^Chi square test

^§^Fishers exact test

^*^Significant (p < 0.050)

According to the results of abdominal ultrasound, out of the 74 diabetic children; 46 (62.2%) had fatty liver (NAFLD) and the rest of them; 28 (37.8%) had normal liver. According to these U/S findings cases were divided into two groups; patients with NAFLD and patients with normal liver, then they were compared together.

This comparison revealed statistically significant difference in age (P < 0.001), the NAFLD group patients were older in age than patients with normal liver (15.4 ± 2.3 vs. 12.4 ± 3.1) year. Regarding patients gender the number of females (63%) in cases with NAFLD was significantly more than those (28.6%) in the group without NAFLD (p < 0.05) as shown in (Table 1).

Concerning the age of onset of diabetes; it was significantly older in cases with NAFLD as compared to cases with normal liver (8.7 ± 3.0 vs. 6.8 ± 3.2) year with (p < 0.05). Moreover, cases with NAFLD had significantly more frequent diabetic complications than cases with normal liver (76.1% vs. 46.4%) with (p < 0.05), including; Lipodystrophy and Neuropathy (p < 0.05) for both. Additionally, cases with liver symptoms were significantly greater in NAFLD patients than those among normal liver group (80.4% vs. 50.0%) with (p < 0.05) especially cases with Abdominal pain (71.7% vs. 42.9%), Nausea (52.2% vs. 21.4%) and Vomiting (41.3% vs. 17.9%) with (p < 0.05) for all. These data are demonstrated in (Table 2).

Regarding the anthropometric characteristics; The mean BMI—Z scores ± SD of patients with fatty liver was 0.45 ± 0.77, no patients with fatty liver were wasted while 29 (63%) of them had normal BMI. Among the 28 cases with normal liver U/S, 2 cases (7.1%) were malnourished (BMI-Z scores ≤ -2.0) while 18 (64.3%) of them had normal BMI (BMI—Z score = 0.0).

Cases with fatty liver as compared to cases with normal liver significantly had higher mean BMI-Z score, (p = 0.001). There was significant difference in proportions of patients according to different BMI—Z scores grades between NAFLD cases and those with normal liver (p = 0.003) with more frequent BMI Z-score grade =  + 1.0** (**32.6% vs. 7.1%) (p < 0.05). On the other hand, waist/ hip ratio and waist/height ratio were significantly increased in the NAFLD patients (p = 0.001) who also had significantly higher sum of skin fold thicknesses than the normal liver group (p < 0.001), (Table 3).

**Table 3:** Blood pressure and anthropometric measurements among the studied cases and comparison according to NAFLD presence

Variables		Total cases (N = 74)	NAFLD		P-value
			Present (N = 46)	Absent (N = 28)	
SBP (mmHg), Mean ± SD		109.9 ± 8.4	109.3 ± 7.4	110.7 ± 9.8	^0.499
DBP (mmHg), Mean ± SD		73.2 ± 5.1	73.0 ± 5.1	73.4 ± 5.1	^0.776
BMI Z-score, Mean ± SD		0.16 ± 0.95	0.45 ± 0.77	− 0.31 ± 1.04	^0.001*
BMI Z-score grades	** ≤ **− **2.0**	2 (2.7%)	0 (0.0%)	2 (7.1%)	#0.003*
	− **1.0**	10 (13.5%)	2 (4.3%)	8 (28.6%)	
	** ± 0.0**	47 (63.5%)	29 (63.0%)	18 (64.3%)	
	** + 1.0**	17 (23.0%)	15 (32.6%)	2 (7.1%)	
	** ≥ + 2.0**	0 (0.0%)	0 (0.0%)	0 (0.0%)	
Waist-hip ratio, Mean ± SD		0.85 ± 0.06	0.87 ± 0.06	0.82 ± 0.05	^0.001*
Waist-height ratio, Mean ± SD		0.46 ± 0.07	0.48 ± 0.07	0.43 ± 0.07	^0.001*
Sum of SFT (mm), Mean ± SD		46.3 ± 17.7	53.8 ± 16.4	34.0 ± 12.2	^ < 0.001*

*SBP* systolic blood pressure, *DBP* diastolic blood pressure, *BMI* body mass index, *SFT* skinfold thickness

^Independent t-test

^#^Chi square test

^§^Fishers Exact test

^*^Significant (p < 0.05)

### The Laboratory findings were as follows

Considering the CBC findings; cases with fatty liver significantly had lower Hemoglobin, RBC count, Hematocrit value compared to cases with normal liver (p = 0.003, 0.037 and 0.005) respectively, while no significant difference between the two groups in the other blood parameters was detected (p > 0.05).

Regarding the lipid profile, the mean serum concentrations of total cholesterol, triglycerides, and LDL were statistically significantly higher in the NAFLD group compared to the other group (p < 0.001, p = 0.019, p = 0.001, respectively). Nevertheless, HDL serum levels were significantly lower in NAFLD cases (p = 0.001).

Comparing the glycemic control; FBG levels were significantly higher among the NAFLD group of patients (p = 0.007). While HbA1c % levels measured at the time of the study and the mean HbA1c % levels during the last year revealed a statistically significant difference among the two groups (p = 0.001 and 0.003 respectively) being higher in the NAFLD group. The number of cases with HbA1c % levels and means > 10% measured at the time of the study and during the last year was significantly higher in the NAFLD group (p = 0.014 and 0.002) respectively.

With respect to the liver enzymes, serum levels of AST and ALT were significantly higher in NAFLD cases compared to normal liver group (p = 0.019 and 0.015). We considered the level of AST > 35 IU/l in males and > 31 IU/l in females abnormal, while ALT > 45 IU/l in males and > 34 IU/l in females was considered abnormal; it was detected that the number of cases with elevated AST serum levels was significantly increased in NAFLD patients compared to cases with normal liver (19.6% vs. 0.0%) with (p = 0.011) while no significant difference was found regarding the number of patients with elevated ALT (p = 0.285). These findings are shown in (Table 4).

**Table 4:** Laboratory findings among the studied cases and comparison according to the presence of NAFLD

Variables	Total cases (N = 74)	NAFLD		P-value
		Present (N = 46)	Absent (N = 28)	
Hemoglobin	13.1 ± 1.4	12.8 ± 1.3	13.7 ± 1.2	^0.003*
RBCs	4.8 ± 0.5	4.7 ± 0.5	4.9 ± 0.4	^0.037*
Hematocrit	38.8 ± 4.1	37.7 ± 4.1	40.5 ± 3.6	^0.005*
MCHC	33.9 ± 0.9	33.9 ± 1.1	34.0 ± 0.5	^0.627
TLC	12.3 ± 5.6	12.0 ± 6.0	12.9 ± 5.0	^0.533
Platelets	354.4 ± 84.9	364.7 ± 86.5	337.5 ± 81.1	^0.184
Cholesterol	148.3 ± 43.2	161.7 ± 45.2	126.4 ± 28.8	^ < 0.001*
Triglycerides	117.1 ± 74.8	132.9 ± 88.8	91.1 ± 29.6	^0.019*
HDL	52.6 ± 8.6	50.2 ± 7.1	56.7 ± 9.4	^0.001*
LDL	71.1 ± 31.3	80.7 ± 33.4	55.5 ± 19.5	^0.001*
FBG	216.2 ± 75.6	234.5 ± 76.8	186.1 ± 64.1	^0.007*
HbA1c	10.3 ± 2.0	10.9 ± 1.7	9.3 ± 2.0	^0.001*
No. of cases with HbA1c ≥ 10.0	40 (54.1%)	30 (65.2%)	10 (35.7%)	#0.014*
Mean HbA1c/year	10.7 ± 2.0	11.2 ± 1.9	9.9 ± 1.8	^0.003*
No. of cases with mean HbA1c ≥ 10.0/year	50 (67.6%)	37 (80.4%)	13 (46.4%)	#0.002*
AST	25.5 ± 18.8	29.5 ± 22.6	19.0 ± 6.1	^0.019*
No. of cases with elevated AST	9 (12.2%)	9 (19.6%)	0 (0.0%)	§0.011*
ALT	14.4 ± 8.5	16.2 ± 10.1	11.3 ± 3.2	^0.015*
No. of cases with elevated ALT	3 (4.1%)	3 (6.5%)	0 (0.0%)	§0.285

^Independent t-test

^#^Chi square test

^§^Fishers exact test

^*^Significant (p < 0.05)

In the context of evaluation of liver fibrosis; (Table 5) shows different stages of fibrosis detected by ARFI elastography in comparison to NAFLD diagnosed by hepatic U/S. The majority of type1 diabetic patients had stage 1 (45.9%), a few had stage 2 (7%) or 3 (5.4%), and one case (1.4%) had stage 4 fibrosis. The proportions of cases with liver Fibrosis detected by ARFI ranging from F2-F4 were significantly more in fatty liver cases than normal liver cases detected by U/S, while the proportion of cases with normal liver (F0) detected by ARFI was significantly higher in cases with U/S normal liver (53.6% vs. 28.3%).

**Table 5:** Different stages of fibrosis diagnosed by (ARFI) compared to NAFLD diagnosed by abdominal ultrasonography

Fibrosis by ARFI	All cases (N = 74) (%)	NAFLD by US Present (N = 46) (%)	NAFLD by US Absent (N = 28) (%)	P-value
F0	28 (37.8)	13 (28.3)	15 (53.6)	§0.021*
F1	34 (45.9)	21 (45.7)	13 (46.4)	
F2	7 (9.5)	7 (15.2)	0 (0.0)	
F3	4 (5.4)	4 (8.7)	0 (0.0)	
F4	1 (1.4)	1 (2.1)	0 (0.0)	

^§^Fisher’s exact test

*Significant (p < 0.05)

Comparison of cases regarding the presence of liver fibrosis delineated that cases with liver fibrosis had significantly higher Cholesterol, Triglycerides and ALT levels compared to cases without liver fibrosis (p < 0.05). As well as significantly had more frequent Abnormal AST (p < 0.05) as shown in (Table 6).

**Table 6:** Laboratory findings among the studied cases and comparison according to liver fibrosis

Variables	Fibrosis		P-value
	Present (N = 46)	Absent (N = 28)	
Hemoglobin	13.0 ± 1.3	13.4 ± 1.4	^0.193
RBC	4.7 ± 0.5	4.9 ± 0.5	^0.219
Hematocrit	38.2 ± 4.1	39.7 ± 4.0	^0.118
MCHC	34.0 ± 0.8	33.7 ± 1.0	^0.219
TLC	12.4 ± 5.6	12.2 ± 5.6	^0.840
Platelets	359.8 ± 83.8	346.1 ± 87.5	^0.504
Cholesterol	157.5 ± 45.8	134.1 ± 35.0	^0.022*
Triglycerides	130.8 ± 90.8	95.9 ± 29.3	^0.020*
HDL	53.5 ± 9.5	51.3 ± 7.0	^0.296
LDL	75.2 ± 34.9	64.8 ± 23.9	^0.164
FBG	214.0 ± 79.3	219.6 ± 70.7	^0.762
HbA1c	10.2 ± 1.7	10.3 ± 2.4	^0.881
No. of cases with HbA1c ≥ 10.0	24 (52.2%)	16 (57.1%)	#0.877
Mean HbA1c/y	10.7 ± 1.8	10.8 ± 2.2	^0.702
No. of cases with mean HbA1c ≥ 10.0/y	30 (65.2%)	20 (71.4%)	#0.837
AST	28.7 ± 23.1	20.6 ± 6.1	^0.069
No. of cases with abnormal AST	9 (19.6%)	0 (0.0%)	§0.010*
ALT	16.1 ± 10.3	11.7 ± 3.2	^0.028*
No. of cases with abnormal ALT	3 (6.5%)	0 (0.0%)	§0.275

^Independent t-test

^#^Chi square test

^§^Fishers exact test

^*^Significant (p < 0.05)

Our results demonstrated that liver fibrosis stage was significantly positively correlated with BMI Z-score, Waist-Hip ratio, Waist-Height ratio, SFT, Cholesterol and Triglycerides levels (p < 0.05) as presented in (Table. 7).

**Table 7:** Correlations of liver fibrosis stages detected by ARFI among the studied cases with other characteristics

Variables	r	p
Age	0.075	0.527
Age of onset	0.182	0.120
Duration	− 0.029	0.806
Insulin dose	0.128	0.276
DKA Frequency	0.021	0.856
SBP	− 0.151	0.198
DBP	0.095	0.421
BMI Z-score	0.339	0.003*
Waist-Hip ratio	0.320	0.005*
Waist-Height ratio	0.411	< 0.001*
SFT	0.411	< 0.001*
Hemoglobin	− 0.134	0.254
RBC	− 0.138	0.240
Hematocrit	− 0.177	0.131
MCHC	0.078	0.510
TLC	− 0.042	0.722
Platelets	0.060	0.611
Cholesterol	0.251	0.031*
Triglycerides	0.244	0.036*
HDL	0.127	0.280
LDL	0.145	0.218
FBG	− 0.023	0.846
HbA1c	0.027	0.818
Average HbA1c	− 0.014	0.905
AST	0.222	0.05
ALT	0.209	0.07

Spearman correlation test

*Significant (p < 0.05)

We investigated the factors associated with occurrence of Fatty liver in type 1 diabetic children and adolescents using Logistic regression models and the BMI-Z score ≥  + 1.0 and Age ≥ 15 years were significant factors that increased the risk of fatty liver occurrence (p = 0.034 and 0.002) with CI (1.140–26.204 and 1.657−9.598) respectively. While being a male was a significant protective factor (p = 0.020) as shown in (Table 8).

**Table 8:** Logistic regression models for factors associated with Fatty liver in T1DM children and adolescents

Factors	Β	SE	P	OR (95% CI)
BMI—Z score ≥ + 1.0	1.699	0.800	0.034*	5.466 (1.140 − 26.204)
Age ≥ 15 years	1.383	0.448	0.002*	3.988 (1.657 − 9.598)
Male sex	− 0.984	0.422	0.020*	0.375 (0.164 − 0.854)

*β* regression coefficient, *SE* Standard error, *OR* Odds ratio, *CI* Confidence interval, *P* is significant at < 0.05

## Discussion

The definition of NAFLD necessitates the confirmation of hepatic steatosis, whether by imaging or by histology, with absence of other reasons for secondary fat infiltration of the liver namely; excessive alcohol consumption, administration of drugs inducing steatosis or genetic diseases [22].

The ‘‘gold standard’’ for diagnosis of NAFLD is Liver biopsy. Nonetheless, it is invasive and has the possibility of complications [23]. Ultrasound is the preferred first-choice imaging method in clinical management [8]. Thus in the present study diagnosis of NAFLD was based on abdominal ultrasonographic findings.

The prevalence of NAFLD among apparently healthy young Egyptian adults aged 19–21 year was studied by Tomah et al. who concluded that fatty liver was present in 31.6% of them [24].

The major finding of our study was the increased prevalence of NAFLD in children with type 1 diabetes as among the 74 patients; 46 (62.2%) cases had NAFLD as diagnosed by abdominal ultra sound (US).

Whereas, Al-Hussaini et al. detected hepatic affection in 10% of 106 children with type 1 diabetes in an Indian study [25], and El-Karaksy et al. (a larger study of 692 Egyptian children with type 1 diabetes) declared a prevalence of 4.5% of liver affection [26]. On the other hand, Farhan et al., reported abnormal hepatic findings in (26%) of children with type 1 diabetes [7] and ElBaki et al. in their study detected 37.3% of cases with NAFLD [27]**.** The high prevalence of hepatic affection in our study could be attributed to poorer glycemic control of the included patients.

Regarding NAFLD patients, they were significantly older in age than patients with normal liver (p = 0.015). This disagrees with the study of Barros et al. [28].

The number of females (63%) in the NAFLD group was significantly more than those (28.6%) in the group with normal hepatic US. On the other hand the percent of females (63%) with NAFLD was more than males (37%) in the same group. While in Farhan et al., study; patients with fatty liver (69.2%) of them were females and in El-Karaksy et al. the female to male ratio was equal. Whereas in Barros et al., study, the female gender represented 75% of the NAFLD group vs. 55.4% in the normal liver group but this was not statistically significant [7, 26, 28].

Moreover, coherent with our results, Samuelsson et al., in their large population study; exihibited a sex difference; girls had poorer metabolic control, i.e., elevated HbA1c concentrations [29]. This could be because girls have poorer metabolic control over the period of adolescence than boys. The variations in hormones might be the influencers among the two genders during this period. Various researches have confirmed that both insulin doses and HbA1c levels were significantly elevated in girls [30, 31].

In examination of children, body mass index (BMI) is one of the most frequently used indicators in evaluation of obesity and undernourishment [32]. The waist-hip ratio (WHR) permits defining the kind of body contour and site of fat accumulation. Waist to height ratio (WHtR) is used to evaluate the dissemination of abdominal adiposity. In pediatric population with abdominal obesity and an amplified risk of metabolic syndrome, the index value is > 0.5 irrespective of sex [33].

Evaluation of anthropometric measures in diabetic children and adolescents must be done regularly. Approaches for nutritional evaluation are harmless and non-invasive, and the study outcomes may be used by clinicians in individuals with diabetes, assisting in monitoring their metabolic control, that affects the appropriate physical growth of children. Therefore, adjusting the nutritional state is of great importance as a whole, and not only stature and weight individually [31].

In this aspect, the anthropometric assessment in the present study revealed that, cases with NAFLD had higher BMI Z scores, waist/hip, waist/ height ratios and sum of skinfold thicknesses than those with normal liver and the difference was statistically significant (p < 0.05). This is in contrast to the findings of Barros et al. [28].

Moreover, our results revealed that cases with NAFLD had more frequent BMI Z-score grade =  + 1.0 (32.6% vs. 7.1%). While**,** 63% of NAFLD cases had normal mean BMI (Z-score = 0) vs. 64.3% of cases with normal liver. None of our patients had obesity (BMI Z score ≥  + 2.0). On the other hand, in the NAFLD group no cases with malnutrition were detected, whereas, 2 cases (7.1%) with normal liver had malnutrition (BMI Z score ≤ − 2.0).

In contrast to our results, Farhan et al. in their study reported; (61.5%) of children with fatty liver had malnutrition while (38.5%) of those children were normal in BMI, in children having normal hepatic findings, (67.6%) were undernourished though (32.4%) had normal BMI [7]**.**

Regarding the lipid profile, our study revealed that patients with NAFLD showed significantly more serum lipid levels (cholesterol, triglyceride and LDL) (p = 0.001, 0.019 and 0.001) respectively, while there was significant decrease in HDL (p = 0.001). This agrees with the findings of previous studies [7, 34, 35].

In acceptance with our results, the findings of Barros et al. showed that cases with altered hepatic US findings had significant elevated triglycerides values and lesser HDL than normal liver patients (p = 0.028 and 0.034) respectively. However, Barros et al. in their study; found that there was no significant difference regarding total cholesterol and LDL levels between cases with Abnormal hepatic US findings and normal liver group [28]. This is opposite to our results.

Oscillations in blood glucose and insulin levels are significant factors in hepatic steatosis associated with type1 diabetes. HbA1c is a good indicator of metabolic control [36]. Proper metabolic control is necessary not only for adequate growth and development in diabetic children and adolescents, but also for reduction and delay of advancement of present complications [37].

Considering mean HbA1c % levels, the mean level of HbA1C of NAFLD patients enrolled in this study was (11.2% ± 1.9) while for those with normal ultrasound findings it was (9.9% ± 1.8) with significant difference (P = 0.003) being higher in the NAFLD group. The number of patients with poor glycemic control as indicated by HbA1c% > 10 was also significantly higher in the NAFLD group.

Parallel to this is the study of Farhan et al. who reported mean value of HbA1C of diabetic patients with fatty liver (10.69 ± 1.41) whereas in normal U/S cases it was (8.24 ± 2.04) with significant difference (P = 0.021) [7]. However, in El-Karaksy et al. study, the mean value of HbA1C in T1D children having liver affection was (8.1 ± 1.2) while for patients with normal liver was (7.6 ± 1.7) with insignificant difference (P = 0.05) [26].

Additionally, in Ismail et al. study, a statistically significant difference in HbA1c % mean levels, among the studied groups (*p* < 0.001) being higher in the NAFLD group with mean level = 8.41% ± 0.8 [35]. This shows that NAFLD children in our study had more improper glycemic control than the previous studies.

Even though assessment of ALT serum level is usually done as a measure of liver functions, its significance is debatable [38]. The dependence on normal liver enzymes is one of the main causes for missing the diagnosis of NAFLD by general practitioners and diabetologists [39].

In this regards, the current study revealed that AST and ALT were statistically significantly higher in patients with NAFLD (P = 0.019 and 0.015). AST > 35 IU/l was considered abnormal in boys and > 31 IU/l in girls; 9 patients only had high level (12.2% of the total cases and they represented only 19.6% of the NAFLD cases). On the other hand ALT > 45 IU/l was considered abnormal in boys and > 34 IU/l in girls; only 3 patients had increased level (4.1% of the total number of cases and they represented only 6.5% of the NAFLD cases) this agrees with the opinion that normal liver enzymes do not exclude fatty liver [39], and further suggests that serum liver enzymes are good indicators for NAFLD diagnosis, however, ‘‘normal’’ standard levels used for exclusion of NAFLD are needed to be reviewed.

Our results agrees with Farhan et al. regarding AST levels, meanwhile, they disagree with them concerning ALT levels [7]. However, Ismail et al. in their study found that the mean serum ALT level of the NAFLD cases was at a high normal level and only three female patients had mildly elevated ALT level [35]**.** Moreover, our results are contradicting to the results of Barros et al. study, [28] and Singh et al. [10]. In addition, several researches demonstrated that the complete histological picture of NAFLD may be detected in patients with normal ALT levels [41, 42].

Diabetic individuals with NAFLD are highly prone to progress into more severe stage of NAFLD which can lead to liver cirrhosis and finally liver failure [8]. Diabetes had been detected as an independent risk factor for hepatic fibrosis [42].

Ultrasound can assess increase in liver size or diffuse increase in hepatic parenchyma echogenicity however it can’t detect fibrosis. ARFI elastography has the privilege that it is not an invasive technique to evaluate liver fibrosis [35].

ElBaky et al. concluded that it is essential to do abdominal ultrasound in type I diabetic pediatric patients as an early non-invasive evaluation of liver affection, whereas ARFI is required in more progressive stages [27]. Moreover, Farhan et al., concluded that NAFLD is significant as an early alarming sign of future result of diabetes mellitus in the form of progression to hepatic fibrosis, cirrhosis and failure [7]

Another major finding in our study is different stages of liver fibrosis diagnosed by ARFI Fibro-Scan compared to abdominal ultrasonography findings. Most of our studied type1 diabetic children and adolescents were having grade one fibrosis (n = 34), a few were with grade 2 (n = 7) or 3 (n = 4), and only 1 patient was having grade 4 fibrosis while 28 patients had F0 stage i.e. no fibrosis.

There was a significant difference between proportions in liver affection using ARFI and those diagnosed by US. From the NAFLD free cases diagnosed by US there was liver stiffness (fibrosis) stage F1 in 13 cases (46.4%) indicating that ARFI can detect liver affection more accurately than US.

Moreover, the number of cases with liver Fibrosis detected by ARFI ranging from F2-F4 was significantly higher among cases with fatty liver compared to cases without fatty liver detected by US (p = 0.021). There was a significant difference between proportions in liver affection using ARFI and those diagnosed by US. 53.6% of the NAFLD free cases diagnosed by US had no fibrosis (F0), while no cases with F2, 3 and 4 were detected in this group. Meanwhile, in NAFLD cases, 28.3% had no fibrosis (F0) and only 26% had intermediate and severe form of fibrosis (F2, F3 and F4).

Ismail et al. in their study; stated that, ARFI elastography classification of fibrosis exhibited that 4 children (8.0%) were having liver fibrosis stage 3 and 4 [35]. While in our study; 10.8% of NAFLD cases had stage 3 and 4 liver fibrosis. Moreover, ElBaki et al. reported ARFI results in 7.7% of patients with different stages of fibrosis [27].

Our results disagree with the results of Farhan et al. [7]. However their results were obtained by calculating the NAFLD fibrosis score and not by fibroscan. Furthermore, a research done by Singh et al. examining 4899 T1D patients aged 18–80 year with suspected NAFLD; showed a prevalence of advanced fibrosis of 22.1% using AST/ALT > 1.4, demonstrating increased risk to develop progressive hepatic affection and its associated complications [10].

On the other hand, previous paediatric studies have shown ARFI to be an accepted non-invasive method. Hanquinet et al. compared ARFI values in children with biopsy-proven chronic liver disease and normal subjects and its value to differentiate between mild and severe (F > 2) fibrosis [43]. In contrast, Kummur et al. did not show any significantly increased prevalence of NAFLD in a paediatric cohort using ALT, ultrasound, and liver stiffness measures [44].

In addition, the findings of Barros et al. showed that of the total participants (8.4%) had significant fibrosis (> F2). Whereas, F2 cases were 3.1%, F3 was present in 3.1% and F4 was present in 2.1% as detected by transient elastography (TE) [28]**.**

Tuong and Duc reported 100% successful rate of ARFI in their study and SWV had significant correlation with degree of liver fibrosis (p < 0.05). They concluded that ARFI was significantly better than APRI in evaluating the degree of liver fibrosis [45].

The current study revealed that Cases with liver fibrosis had significantly higher Cholesterol, Triglycerides and ALT serum levels compared to cases without liver fibrosis (p < 0.05). Cases with Abnormal AST serum level were significantly more frequent among the liver fibrosis group (p < 0.05).

Compatible with our study, Carter-Kent et al. mentioned that, the serum AST level correlated with the stage of fibrosis and might be used to differentiate significant from no or mild fibrosis [46]. In agreement with our findings also Farhan et al. reported that, AST serum level was found a good predictor of fibrosis in pediatric patients (P-value = 0.001) [7].

We analyzed factors correlated with the stage of liver fibrosis in the present study and we detected significant positive correlations of Liver fibrosis stages with BMI Z-score, Waist-Hip, Waist-Height, SFT, Cholesterol, Triglycerides (p < 0.05).

Multivariate logistic regression was applied to assess risk factors associated with occurrence of NAFLD; BMI-Z score ≥  + 1.0 and Age ≥ 15 years were detected as significant factors associated with increased risk of fatty liver development. While being a male was a significant protective factor.

In Barros et al. study; multivariable logistic regression assessing associated factors with fatty liver using both imaging technique; Gender, age and HbA1c were not associated to steatosis. This is contradicting to our results where we found gender and age as associated risk factors for NAFLD occurrence. However, Barros et al. study revealed that triglycerides were the only risk factor for fatty liver [28]**.** This could not be detected in our study.

However, Sae-wong et al. found that high BMI-SDS were the only risk factor associated with NAFLD (OR, 5.79) [47]. This coincides partly with our results.

## Limitations of the study

Our study has some limitations. First, we diagnosed NAFLD based on ultrasound which is operator-dependent and has a limited sensitivity. Second, ARFI fibroscan; the second technique we used, is not commonly used as first- choice investigation for diagnosis of liver fibrosis. Fibro—test consists of a panel of markers for diagnosis of liver fibrosis but unfortunately we couldn’t do it as we didn’t have enough fund for these tests which would cost too much so we didn’t perform them. We have used the ARFI fibroscan to diagnose Liver fibrosis according to previous studies which evaluated the diagnostic performance of ARFI fibroscan in diagnosing liver fibrosis [45, 48–51]. Thirdly, we did not have histological confirmation of our findings as the gold-standard method; liver biopsy, is invasive and prone to sample mistakes. Another limitation was the small sample size of the study.

As strengths in our study; NAFLD was diagnosed using two methods in this sample of type1 diabetic patients while most of NAFLD studies in type1diabetes used ultrasonography only. In addition, to the best of our knowledge, little previous studies were performed to diagnose steatosis and assess hepatic fibrosis in type1 diabetes children thus this data are represented for Egyptian children.

## Conclusions

The primary outcome of this study demonstrated a very high prevalence of NAFLD in T1D children and adolescents using US which was (62.2%) and the percent of significant and advanced liver fibrosis (F2-F4) in NAFLD cases using ARFI elastography was 26%. The study clarified that, 46.4% of the NAFLD free cases detected by US had mild liver fibrosis (F1) as detected by ARFI.

NAFLD represents a serious consequence of type 1 Diabetes in children and adolescents. It is an early warning sign of future consequence of diabetes mellitus in the form of progression to liver fibrosis, cirrhosis and failure; especially in those with poor glycemic control. NAFLD has the potential to evolve to fibrosis. Ultrasound can detect hepatomegaly and diffuse increase in hepatic parenchyma echogenicity but not fibrosis. ARFI fibro-scan assessment of liver fibrosis in T1DM patients with NAFLD detected the presence of significant fibrosis. BMI, age of patients and female gender were among the risk factors associated with NAFLD. Further prospective studies with larger number of patients are recommended.

## Data Availability

The datasets used and/or analyzed during this study are available from the corresponding author on reasonable request.

## Abbreviations

ALT: Alanine transaminase
ASMA: Anti-smooth muscle antibodies
LKM: Anti-liver and kidney microsomal antibodies
ANA: Anti-nuclear antibodies
ARFI: Acoustic radiation force impulse
AST: Aspartate transaminase
BMI: Body mass index
BP: Blood pressure
CBC: Complete blood count
CVD: Cardiovascular disease
DBP: Diastolic blood pressure
FBG: Fasting blood glucose
Hb: Haemoglobin
HbA1c: Glycosylated haemoglobin
HBVs Ag: Hepatitis B virus surface antigen
HCV Ab: Hepatitis C virus antibodies
HDL: High density lipoproteins
IDF: International diabetes federation
ISPAD: International Society for paediatric and adolescent diabetes
K: Korotkoff sound
LDL: Low density lipoproteins
MCHC: Mean corpuscular haemoglobin cocentration
NAFLD: Non-alcoholic fatty liver
NASH: Non-alcoholic steato-hepatitis
RBCs: Red blood cells
ROI: Region of interest
SBP: Systolic blood pressure
SD: Standard deviation
SKF: Skinfold thickness
SPSS: Statistical package for social sciences
T1DM: Type1 diabetes mellitus
TE: Transient elastography
TLC: Total leucocytic count
U/S: Ultrasound
Vs: Versus
WHO: World Health Organization
WHR: Waist to hip ratio
WHtR: Waist to height ratio
